# Impact of Preheating Temperature on the Separation of Whey Proteins When Combined with Chemical or Bipolar Membrane Electrochemical Acidification

**DOI:** 10.3390/ijms21082792

**Published:** 2020-04-17

**Authors:** Claudie Aspirault, Alain Doyen, Laurent Bazinet

**Affiliations:** 1 Department of Food Sciences, University Laval, Québec, QC G1V 0A6, Canada; claudie.aspirault.1@ulaval.ca (C.A.); alain.doyen@fsaa.ulaval.ca (A.D.); 2 Laboratoire de Transformation Alimentaire et Procédés ÉlectroMembranaires (LTAPEM, Laboratory of Food Processing and ElectroMembrane Processes), University Laval, Québec, QC G1V 0A6, Canada; 3 Institute of Nutrition and Functional Foods (INAF), Dairy Research Centre (STELA), University Laval, Québec, QC G1V 0A6, Canada

**Keywords:** whey proteins, α-lactalbumin, β-lactoglobulin, eco-efficient process, proteins separation, electrodialysis with bipolar membrane, heating temperature

## Abstract

Separation of α-lactalbumin and β-lactoglobulin improves their respective nutritional and functional properties. One strategy to improve their fractionation is to modify their pH and ionic strength to induce the selective aggregation and precipitation of one of the proteins of interest. Electrodialysis with bipolar membrane (EDBM) is a green process that simultaneously provides acidification and demineralization of a solution without adding any chemical compounds. This research presents the impact on whey proteins separation of different preheating temperatures (20, 50, 55 and 60 °C) combined with EDBM or chemical acidification of 10% whey protein isolate solutions. A β-lactoglobulin fraction at 81.8% purity was obtained in the precipitate after EDBM acidification and preheated at 60 °C, representing a recovery yield of 35.8%. In comparison, chemical acidification combined with a 60 °C preheating treatment provides a β-lactoglobulin fraction at 70.9% purity with a 11.6% recovery yield. The combination of EDBM acidification with a preheating treatment at 60 °C led to a better separation of the main whey proteins than chemical acidification.

## 1. Introduction

Cheese whey is an important source of proteins with interesting nutritional and functional properties [1,2,3]. The two major whey proteins, β-lactoglobulin (β-lg) and α-lactalbumin (α-lac), represent 50% and 20%, respectively, of the total whey protein content [4]. The protein β-lg is widely used in the food industry due to its emulsifying, gelling and foaming properties [5,6]. Infant formula is supplemented with α-lac due to its crucial role in infant development related to its amino acid composition [4,7]. However, β-lg is a main allergen in formula produced from bovine milk and needs to be eliminated. 

Consequently, different strategies were published to specifically fractionate β-lg and α-la. However, the separation of those two proteins is challenging due to their similar molecular weights and isoelectric points. Ion-exchange chromatography is one of the principal methods used for whey protein fractionation [1,8,9]. It is in fact the most efficient method, but it also has a very high cost [1]. Thermal treatment and/or pH adjustment were also applied [10,11,12], as was metaphosphorus complex precipitation [13]. Recently, a new method was proposed by Marciniak et al. that combines acidification and high hydrostatic pressure [14]. Pressurization of 600 MPa for 5 min allows reaching an α-lac purification rate of 86% with recovery yield of 77%. Thermal treatment can be used to separate α-lac and β-lg due to their different denaturation temperatures of 65 and 75 °C, respectively [10,11,12]. Despite this fact, α-lac is more thermally stable than β-lg due to the presence of the calcium ion in its core [10]. Adding thermal treatment to acidification can lead to aggregation and precipitation of one of the major proteins [10]. Other separation methods have been developed using membrane processes like ultrafiltration alone [15] or combined with electrodialysis [16]. This last method proposes the demineralization of a whey protein concentrate by conventional electrodialysis combined with a chemical acidification until a final pH of 4.65. This method led to the production of a fraction that contains 17% initial whey proteins. These methods allow protein separation based on molecular weight and/or isoelectric point in a less expensive way than chromatography [1], but the presence of fouling on the membrane reduces the process efficiency [17,18]. However, all those methods allowing the separation of β-lg and α-la require the use of chemical compounds in order to complete the acidification step and/or to elute the compounds for the chromatography. To the best of our knowledge, there is no process that allows selective separation of the two major whey protein without adding a chemical compound in the solution of interest during the acidification step. 

Electrodialysis with bipolar membrane (EDBM) is an eco-efficient process [19] that does not require the use of chemical compounds, except for the cleaning step, because acidification is obtained by bipolar membrane which, under an electric field, dissociates water molecules into protons and OH^–^ ions [20]. Due to the stacking of ion-exchange membranes in the EDBM cell configuration, EDBM enables simultaneous acidification and demineralization of the solution [21]. This method has already been used for the separation of casein from milk in a way that is less harmful to the environment than processes requiring the use of chemicals [17,19]. In 2004, Bazinet et al. studied the possibility to fractionate whey proteins by EDBM for the acidification step [21]. Their studies have shown that by using a 10% whey protein solution from a whey protein isolate powder, it was possible to get a 53.4% recovery yield of β-lg with a purity rate of 97.3% in the precipitate. In addition, studies focusing on fractionation of whey protein using electrodialysis showed the impact of protein concentration, pH and electrical conductivity of the solution on the precipitation and separation of whey proteins [16,21,22], but the selective separation of α-lac and β-lg in pure fractions was not yet obtained. However, the denaturation rate combined with EDBM treatment has never been studied. Indeed, preliminary tests have shown a major difference in terms of final fraction purity when two whey protein isolates (WPIs) with different denaturation rates were acidified with the same EDBM system and parameters. 

Therefore, the aim of the present study is to optimize the separation of the two major whey proteins when the acidification step is performed by chemicals, as it is usually obtained in other studies, or by EDBM by adding a preheating step at different temperatures. 

## 2. Results

### 2.1. Electrochemical Acidification Parameters

#### 2.1.1. Duration and System Resistance

The duration to carry out EDBM was the same regardless of the preheating temperature used (*p* = 0.098). For all temperature averaged, the EDBM duration to decrease the pH from 6.6 to 4.8 was 117.9 ± 17.5 min. The EDBM was stopped when the pH of whey protein solution was stabilized at pH 4.8. At this pH value, the system resistance was too important to continue the process. 

In addition, during EDBM, the evolution of the system resistance was also the same regardless of the preheating temperature (*p* = 0.065). The resistance increased exponentially from 18.3 ± 0.6 Ω to a maximum resistance of 132 ± 25 Ω (*p* < 0.001).

#### 2.1.2. Membrane Electrical Conductivity

Table 1 shows the conductivity values of membranes stacked in the EDBM system at different positions before and after acidification. No significant difference was observed in the variation of membrane conductivity between the different temperatures tested (*p* > 0.05) (results not shown). However, there were significant differences between the variation of the conductivity depending on the position of the membrane (*p* < 0.001). Variation of conductivity of AEM was the lowest with an increase of 4.2%, while CEM 2 had the highest variation in conductivity before and after acidification with a decrease of 35%. The BM, CEM2 and CEM3 conductivity values after EDBM treatment were significantly lower than their initial ones (BM: *p* = 0.007; CEM2: *p* < 0.001; CEM3: *p* = 0.009). The AEM conductivity after EDBM treatment was, in contrast, significantly higher than its initial conductivity (*p* = 0.011). 

### 2.2. Whey Protein Solution Parameters: Conductivity and pH Evolution

**Figure 1:**
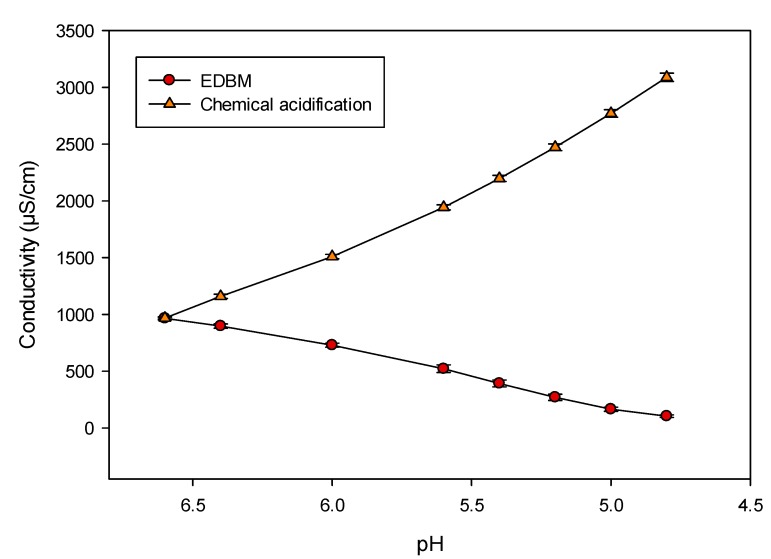
Evolution of whey protein isolate (WPI) solution conductivity during chemical and EDBM acidifications.

Figure 1 shows the evolution of the WPI solution conductivity during both acidification methods. WPI solutions at 10% protein had an averaged conductivity of 965 ± 13 µS/cm (*p* > 0.05). There was a significant difference in the conductivity of WPI solution after acidification between EDBM and chemical acidification (*p* < 0.001). EDBM acidification led to a significant decrease in conductivity, which dropped to 104 ± 11 µS/cm (*p* < 0.001), corresponding to a demineralization rate of 89%. In contrast, the chemical acidification increased the conductivity significantly to 3089 ± 37 µS/cm (*p* < 0.001), corresponding to a mineralization rate of 220%.

### 2.3. Precipitate Analyses 

#### 2.3.1. Precipitate Weights

**Figure 2:**
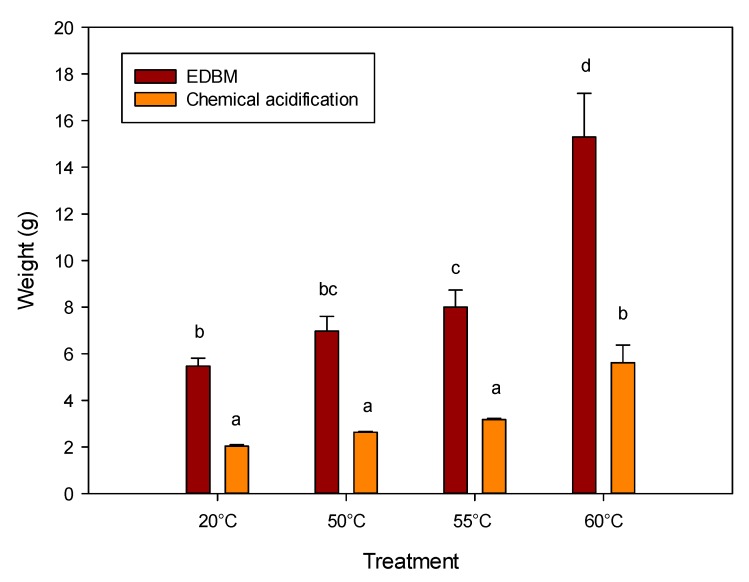
Weight of freeze-dried precipitate fractions generated at pH 4.8 after both acidification methods. The results are presented as the means ± standard deviation of three independent experiments. The use of different lowercase letters (a, b, c, d) indicate significant statistical differences between data.

Figure 2 shows the weight of freeze-dried precipitates generated after both acidification methods. Both temperature and acidification methods had an impact on the weight of precipitate fractions (*p* < 0.001). For the chemical acidification, 60 °C was the only preheating temperature that allows a significant increase in weight of the precipitate obtained from the four other temperatures. However, the weight obtained with the chemical acidification at 60 °C was the same as the lowest weight obtained by EDBM acidification without preheating treatment (*p* = 1.000). For EDBM acidification, the weight of the final precipitate increased as a function of the preheating temperature. The 60 °C preheating temperature combined with EDBM acidification provided the highest weight amongst all conditions (15.3 ± 1.9 g). With that combination of treatment, the weight of the precipitate was 3 times higher than the EDBM acidification at 20 °C and 7 times higher than the combination of chemical acidification and a 20 °C preheating treatment. 

#### 2.3.2. Proximate Composition

Table 2 shows the proximate composition of the freeze-dried control and precipitates. Fat content was similar regardless of the acidification method (*p* = 0.118) and preheating temperature (*p* = 0.245). Lactose content in precipitates obtained with EDBM and chemical acidification methods (all temperature averaged) was significantly lower than that of the control (0.19 and 0.25 vs. 0.77 g/100 g, respectively) (*p* < 0.001). Lactose content in precipitates obtained with EDBM acidification was slightly lower than that of samples obtained with chemical acidification. In fact, there was a statistically significant interaction between the preheating temperature and the method of acidification used (*p* = 0.002). 

Ash content in all the precipitates was lower compared to the control (*p* < 0.001). Moreover, ash content of precipitate was different depending on the acidification method (*p* < 0.001). All the samples obtained by EDBM acidification (all temperatures averaged) had lower ash content than the samples obtained by chemical acidification (0.42 vs. 1.00 g/100 g, respectively). There was no significant difference (*p* > 0.05) between each preheating temperature within EDBM or chemical acidification. Furthermore, the ash content tended to decrease with an increase of the preheating temperature (Table 2) for both acidification methods. Effects of the temperature (*p* < 0.001) and of the acidification method (*p* < 0.001) were observed on the ash content, but no interaction between both factors was observed (*p* = 0.578). 

The calcium, potassium, magnesium, sodium and phosphorus concentrations in the precipitated fractions in comparison with the control are also presented in Table 2. Except for sodium content, all ion species in the precipitates were different from the control regardless of the acidification method or the preheating temperature. Calcium, potassium and magnesium contents were lower in the treated sample than in the control, while phosphorus content was higher in the treated sample. On the other hand, sodium content was not influenced by the acidification method (*p* = 0.218) or the temperature treatment (*p* = 0.117). There was also no interaction between both factors for the sodium content (*p* = 0.124). For calcium, potassium, magnesium and phosphorus, ion contents were lower for the EDBM acidification than for the chemical acidification, regardless of temperature. For the chemical acidification, calcium, potassium and magnesium contents increased with an increase in temperature up to 60 °C. On the other hand, phosphorus content decreased with an increase in temperature (0.460 to 0.333 g/100 g). Ion concentrations for the samples acidified with EDBM were not influenced by the increase in the preheating temperature. Calcium was the major ion present in the control and represented one-third of the total mineral content (0.660 g/100 g). Its concentration dropped by more than half for the chemically acidified samples (0.275 g/100 g, all temperatures averaged) and was 13 times lower for the EDBM-acidified samples (0.049 g/100 g, all temperatures averaged). The calcium content was indeed influenced by the interaction of the type of acidification and the preheating temperature treatment (*p* < 0.001). 

The total protein content was only influenced by the method of acidification (*p* = 0.010). Indeed, chemical acidification led to precipitates with slightly higher total protein content (all temperatures averaged: 97.3 g/100 g for EDBM vs. 98.2 g/100 g for chemical acidification). The only value that was significantly different from the initial protein content was the one obtained for chemical acidification combined with a preheating temperature of 60 °C (*p* = 0.015). However, this value was not significantly different from the other treated samples (*p* > 0.05). 

#### 2.3.3. Protein Yield

**Figure 3:**
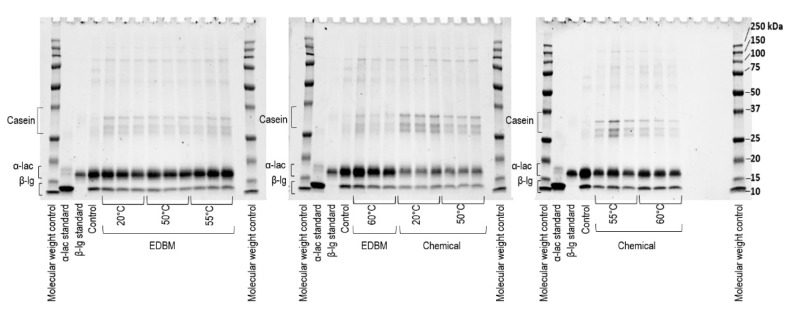
Denatured and reduced PAGE of precipitated fractions recovered after acidification at pH 4.8.

Figure 3 presents protein profiles of precipitates generated at pH 4.8. According to the gels, all the precipitates were mainly composed of β-lg but still had α-lac in a non-negligible way. They were also composed of casein, immunoglobulin, lactoferrin and serum albumin to a lesser extent, as can be seen by the upper bands on the gels. The intensities of the β-lg and α-lac bands were higher for the samples acidified with EDBM than for those chemically acidified, especially for those heated at 20 and 50 °C. In fact, those samples had higher band intensity for casein and lower intensities for β-lg and α-lac than the other samples. The α-lac bands for samples chemically acidified and heated at 55 and 60 °C had higher intensity than those at the same temperatures and acidified by EDBM. Analysis of the α-lac and β-lg bands provided the exact composition value of each of the major whey proteins.

##### Protein Profile

Table 3 shows purity (%), weights of proteins and recovery yield (%) of α-lac, β-lg and total proteins in the precipitate fractions. The β-lg purities of the precipitates obtained with the EDBM acidification were all higher than the control regardless of preheating temperatures (*p* < 0.001). A β-lg purity close to 82% was obtained, in comparison with its initial value of 74.23 ± 0.24% in WPI. The comparison of the control with chemically acidified samples showed no difference with the samples that were preheated at 20, 50 or 55 °C (*p* > 0.05). On the other hand, the combination of chemical acidification with preheating at 60 °C provided a significantly lower β-lg purity than the control (70.94 vs. 74.23%; *p* < 0.001). There was also a statistically significant interaction between the preheating temperature and the acidification method (*p* < 0.001). The β-lg purity obtained with EDBM acidification in the precipitates was higher than those obtained with chemical acidification (*p* < 0.001). Furthermore, the use of a preheating step (50, 55 or 60 °C) combined with EDBM acidification increased significantly the purity of the precipitate in comparison with 20 °C (*p* = 0.002), but there was no significant difference between those three temperatures. For the chemical acidification, β-lg purity decreased with an increase in the preheating temperature (74.12 ± 0.80% to 70.94 ± 0.55%). 

The purity of α-lac in samples acidified with EDBM was lower compared to the control (*p* < 0.001 for all of them), but chemical acidification combined with heating temperature of 20 to 55 °C did not show any difference with the control (*p* > 0.05). Only the 60 °C preheating temperature for the chemical acidification allowed a significant increase of the α-lac purity (*p* = 0.001). The α-lac purity in the precipitate of EDBM acidification was also different from the chemical acidification (*p* < 0.001 for all of them). In addition, for the EDBM acidification, α-lac purity in the precipitate decreased from 21.68 ± 0.68% to 17.75 ± 1.60% between 20 °C and 50 °C and then stabilized for temperatures of 50, 55 and 60 °C. No difference was detected between the three preheating temperatures in terms of α-lac purity (50–55 °C: *p* = 0.999; 50–60 °C: *p* = 1.094; 55–60 °C: *p* = 0.847). For the chemical acidification, α-lac purity increased from 25.88 ± 0.80% at 20 °C to 29.06 ± 0.55% with an increase in the preheating temperature to 60 °C (*p* = 0.003). 

##### Ratio of β-lg/α-lac

Table 3 shows the β-lg/α-lac ratios obtained in the final precipitate fractions. There was a statistically significant interaction between the preheating temperature and the acidification method (*p* < 0.001) of the β-lg/α-lac ratio in the precipitate fraction. For the EDBM acidification, ratios were higher than for the control (20 °C: *p* = 0.003; 50, 55 and 60 °C: *p* < 0.001) while the chemical acidification led to similar ratios as the control at all preheating temperatures (20 °C: *p* = 1.000; 50 °C: *p* = 1.000, 55 °C: *p* = 0.702; 60 °C: *p* = 0.160). For the EDBM acidification, the samples preheated at 50, 55 or 60 °C have ratios 1.6 times higher than the control, and there was no significant difference between the ratios of those three temperatures (*p* < 0.001). Even if there was no significant difference between the ratio of chemically acidified samples, a tendency can be observed: the increase in preheating temperature provided a lower ratio (2.87 vs. 2.44). In the supernatant, the β-lg/α-lac ratio for the EDBM acidification was lower than the control when the preheating temperature was 50 °C (2.88 vs. 2.68 for control and 50 °C respectively), and this ratio decreased as the temperature increased (2.41 for 60 °C). 

##### Protein Recovery Yield

Table 3 shows the recovery yields of β-lg, α-lac and total protein content in the freeze-dried precipitate fractions. The recovery yields of the total protein and the β-lg followed similar tendencies. For the same temperature, recovery yields were all higher for the EDBM acidification than for chemical acidification (20 °C: *p* = 0.003; 50 °C: *p* < 0.001; 55 °C: *p* < 0.001; 60 °C: *p* < 0.001). Regardless of the preheating temperature used, the total protein and the β-lg recovery yields were 2.5 and 2.9 times higher, respectively, with the EDBM than with the chemical acidification. It can also be noted that the highest total protein recovery yield obtained with chemical acidification (with a 60 °C preheating treatment) was statistically the same as the lowest one obtained with EDBM acidification combined with the 20 or the 50 °C treatment (*p* = 0.997 and *p* = 0.497, respectively). The same pattern was observed for the β-lg recovery yield: the highest β-lg recovery yield obtained with chemical acidification (at 60 °C) was statistically the same as the lowest one obtained with EDBM acidification (at 20 °C; *p* = 0.035). For both acidification methods, the total protein and β-lg recovery yields increased with an increase in temperature. The recovery yield was in fact 3 times higher for the 60 °C treatment than that for the 20 °C treatment, for both EDBM and chemical acidification. The maximum total protein and β-lg recovery yields of 32.56 ± 3.96% and 35.78 ± 4.39%, respectively, were obtained for the EDBM acidification combined with a 60 °C preheating treatment.

The α-lac recovery yields were also higher for the EDBM acidification than for chemical acidification at the same temperature (20 °C: *p* = 0.012; 50 °C: *p* = 0.022; 55 °C: *p* = 0.027; 60 °C: *p* < 0.001), but the differences were less important than for the β-lg recovery yield. The α-lac recovery yields with EDBM acidification were the same from 20 to 55 °C and increased at 60 °C. The same phenomenon happened for the chemical acidifications. For the EDBM samples, α-lac recovery yields were lower than the β-lg yields for temperatures 50, 55 and 60 °C (20 °C: *p* = 0.897; 50 °C: *p* = 0.005; 55 °C: *p* < 0.001; 60 °C: *p* < 0.001). For the chemical acidification, the α-lac recovery yields were non-statistically different from the β-lg yields, regardless of the temperature (*p* > 0.05). These results are in accordance with the ratios for EDBM acidification, which showed β-lg precipitation, and the ratios for chemical acidification, which promoted α-lac precipitation. 

## 3. Discussion

The aim of the present study was to optimize the separation of α-lac and β-lg in a whey protein solution when the acidification step was performed by chemicals or by EDBM by adding a preheating step at different temperatures. The condition using EDBM acidification combined with a preheating temperature of 60 °C led to the recovery of a β-lg-enriched precipitate fraction with a purity of 81.75 ± 0.33% and a recovery yield of 35.78 ± 4.38%. Chemical acidification did not lead to one protein-enriched fraction, neither in the precipitate nor in the supernatant. 

### 3.1. Electrochemical Acidification Parameters 

#### 3.1.1. Duration and System Resistance

It has been shown that EDBM duration was protein-dependent due to the number of H^+^ ions to be electrogenerated, which is proportional to the protein concentration [21]. In our case, the protein concentration of all initial solutions was 10%, which can explain why no significant difference was observed in terms of duration between each treatment. The increase in EDBM system resistance can be explained by the demineralization of the WPI solution due to the migration of one cation for every proton generated by the bipolar membrane in a way to maintain electroneutrality. The stabilization of pH value at 4.8 was due to the fact that the major part of the cations were migrated through the CEM2 at the end of the EDBM and, consequently, the generation of one H^+^ ion provokes the migration of another H^+^ ion into the recovery compartment to compensate for the lack of mobile cations. Thereby, acidification cannot occur, and the pH stays the same. This phenomenon was previously explained by Bazinet et al. (2000) [23]. 

#### 3.1.2. Membrane Electrical Conductivity

Since there was no difference between membranes at the same position regardless of the preheating temperature used, the variations in conductivity observed were not influenced by the increase in preheating temperature. The variations observed between each position of membranes can be explained by the difference in ion composition in the solutions that migrated through these membranes. The CEM2 and the BM were the two membranes that separated the whey protein compartment, while the other membranes separated NaCl solutions. The decrease in conductivity observed for the CEMs has already been described by Dufton et al. (2019) [24]. It is due to the transfer of counterions that have lower electrophoretic mobility than the Na^+^ counterions initially present in the membrane. Indeed, calcium and magnesium are two of those counterions that have lower electrophoretic mobility than sodium (Ca^2+^: 1.07 × 10^9^ cm^2^/V·s; Mg^2+^: 0.91 × 10^9^ cm^2^/V·s; Na^+^: 4.39 × 10^9^ cm^2^/V·s) [25] and were also present in the WPI solution [25]. The CEM2 was the membrane that allowed the transfer of cations from the WPI solution into the recovery solution compartment. Therefore, more Ca^2+^ and Mg^2+^ migrated through this membrane than any other membrane of the system, explaining the highest membrane conductivity reduction observed. The slight decrease in conductivity of BM was explained by the presence of ions from the WPI solution that may have passed through it in lower proportion than in the CEM2 instead of protons. Such a decrease in membrane conductivity would also have contributed to the decrease in the global system observed previously. 

### 3.2. Whey Protein Solution Parameters: Conductivity and pH Evolution

As expected, the WPI solutions had the same conductivity values since all the protein solutions were prepared in the same way. The decrease in conductivity of WPI solutions during EDBM treatments was consistent with data reported in the literature [21] and was due to the migration of positively charged species, such as Ca^2+^, Na^+^, K^+^ and Mg^2+^, through the cation-exchange membrane (CEM2) of the WPI solution compartment. Such a decrease in conductivity confirmed the increase in global cell resistance observed previously due to the demineralization of the WPI solution. On the other hand, the chemical acidification increased the conductivity by more than 3 times the initial conductivity, regardless of the preheating temperature used. This increase was due to the addition of HCl, which increases the H^+^ and Cl^–^ ion contents in the solution. As the conductivity of a solution is determined by the ion mobility and concentration [26], such conductivity increase in the WPI solution was expected. Since the volume of HCl required for acidification was the same for each preheating temperature (10.7 mL), the similar final conductivities obtained for all the chemically acidified samples were also expected. 

### 3.3. Precipitates Analyses

#### 3.3.1. Precipitate Weights

The difference observed between the precipitate weights of chemical and EDBM acidification can be explained by the different conductivities in the solutions depending on the acidification method. Higher conductivity generally means higher mineral content that helps to keep WPI content in solution [27]. The use of preheating temperature also led to higher precipitate weights, and this can be due to the denaturation of the proteins that led to their aggregation and precipitation [28]. To confirm these differences in precipitate weights, their compositions were analyzed. 

#### 3.3.2. Proximate Composition

The decrease in ash content between the control and the treated samples was explained by the fact that the control is the initial WPI powder, while the other samples came from the precipitated fractions only. Hence, since ashes represent the total mineral content and these minerals are mostly soluble, minerals tended to stay in the supernatant while only a part precipitated with the proteins [29,30]. The difference between both acidification methods was due to the demineralization phenomenon during EDBM acidification leading to a significant decrease in mineral content. This result confirmed the decrease in conductivity reported previously during EDBM acidification. On the other hand, chemical acidification involved addition of protons and Cl^–^ ions without demineralization. The same results have been observed by Bazinet et al. (2004) [21].

As mentioned previously for ash contents, the difference observed between the calcium, potassium, magnesium and sodium contents of the control and the treated samples was because the treated samples were precipitate fractions. The decrease in calcium, potassium, magnesium and sodium content for the precipitates obtained with the EDBM acidification method was in accordance with the literature. Indeed, potassium is a cation known to be the predominant cation to migrate across the CEM during EDBM acidification, followed by sodium, magnesium and calcium [23]. Even if sodium is supposed to be the second predominant cation to migrate across the CEM, its concentration decrease was lower than the one for the calcium. This difference from the literature can be explained by the different initial concentrations of those two cations. Initial calcium content was in fact 2.5 times higher than the sodium content, and, since the migration of a compound is directly correlated to its concentration (as demonstrated by Aider et al. (2006) [31]), calcium has migrated in a larger proportion than sodium. 

For the sodium content in chemically acidified samples, the differences between control and treated samples were not statistically significant, except at 50 °C when all the samples (EDBM and chemical acidification) were compared to the control. In fact, if only the chemically acidified samples were compared to the control, they were all significantly different from the control and contained less than half of the sodium content (*p* < 0.05 for each of the temperature). The increase in the ion contents with the increase in temperature observed for the chemically acidified samples was explained by the unfolding of α-lac and β-lg in reaction to the preheating treatment leading to negative charges on the proteins more accessible to cations [28]. Thereby, more cations, mainly divalent due to greater possibility of electrostatic interactions, could have precipitated with the proteins. On the other hand, phosphorus is a negatively charged ion that did not pass through the cationic membrane during the EDBM acidification. That explains why the phosphorus content was the highest in terms of concentration after EDBM, while initially calcium was the major ion in the control. Concerning phosphorus, its content was higher in the chemically acidified sample than in the control since phosphorus precipitated more. Since treated sample composition is in g/100 g of lyophilized precipitate while control is in g/100 g of initial WPI, this represents a decrease of 8% of the initial phosphorus content for the 20 °C treatment combined with chemical acidification. Phosphorus is an anion that can promote protein aggregation when combining with NaCl by binding with oppositely charged amino groups of whey proteins, leading to the repulsion between peptide chains and increased exposition of the protein negative charges [32]. Since there was a higher sodium content in chemically acidified samples than those with EDBM acidification, it was expected to obtain higher phosphorus content in the precipitate fraction obtained from chemical acidification method. 

Lactose is a soluble component, and it is generally found in the supernatant of centrifuged samples [33]. In the actual case, precipitates were not washed, which means that it was possible that part of the lactose present in the supernatant remained in the precipitate despite a 10,000× *g* centrifugation. In addition, β-lg has the potential to react with lactose by lactosylation, and that phenomenon is influenced by temperature [34]. This explain why the lactose content tend to increase with the increase of the temperature for both acidifications. 

Since the initial product was a whey protein isolate, it was expected to have low lipid content. WPIs have low levels of lipids, mostly due to the preparation method that removes most of them [35]. Residual lipids present in WPI solution did not interact with protein when there was an acidification or a preheating step up to 60 °C.

Proteins represented the major part of all precipitates, and since the proteins used came from a WPI powder with 96.31% ± 0.11% proteins, this result was expected. The interesting fact here is that the exact protein composition can be different depending on the conditions used.

#### 3.3.3. Protein Yield: Protein Profile, Ratio of β-lg/α-lac and Recovery Yield

##### Effect of pH

According to Amundson et al. (1982) [16], there are three major factors that influence the recovery yield in fractionation process: the ash content, the protein concentration and the pH of the solution. The minimum pH obtained in our study was 4.8 for every condition. This pH was higher than the recommended pH of 4.65 that has been proved to allow a maximum formation of β-lg octamers [36,37,38] and to allow higher recovery yield [16,21]. In Amundson et al.’s (1982) study on the fractionation of enriched fractions of β-lg and α-lac from whey protein concentrate solution, the protein recovery yield was in fact increased by 2.1 times between pH of 5.2 and 4.65 [16]. Bazinet et al. (2004) evaluated the total peak area profile with reverse-phase HPLC of β-lg and α-lac in the supernatant with the same total protein concentration as us (10% protein) when acidified with EDBM at different pH from 6.8 to 4.6 [21]. There was a difference of 9.2 units of percentage between the β-lg total peak area from pH values 4.8 to 4.6, which means that a large part of β-lg precipitated (corresponding to a 18.5% additional precipitation) between those two pH values. The area profile of α-lac only showed a decrease of 0.9 unit of percentage, which means that α-lac does not precipitate as much as β-lg between those two pH values. With this information, it is possible to determine that the minimum pH of 4.8 reached in our study influenced the purity and the recovery yield obtained with our conditions. It will be expected to obtain higher β-lg purity and recovery yield in the precipitated fraction of the WPI solution acidified to pH 4.65 instead of 4.8 with EDBM acidification method. However, the effect of pH is directly dependent on other factors like the mineral content and temperature, and it is the combination of the pH with those factors that can lead to a selective separation of the major whey proteins. 

##### Effect of Mineral Content

Protein profile obtained in the precipitated fraction was influenced by the mineral content of the solution. As presented previously, mineral content depended on the acidification method. EDBM acidification allowed an important demineralization of the WPI solution, while chemical acidification led to an increase in conductivity by HCl addition. 

The differences in protein purity obtained between the control and the acidified samples confirmed that by changing pH and conductivity conditions, it was possible to promote precipitation of one of the major whey proteins. A preheating temperature of 60 °C with EDBM acidification allowed the highest precipitation of the β-lg, but the highest precipitation of α-lac was also obtained. It was also in these conditions that the recovery yield of the total protein was the highest, and that was consistent with the results of Amundson et al. (1982) [16]. In their study, they showed that the demineralization of an acidified whey solution allowed the recovery yield of proteins in the precipitate fraction to increase by 4.5 times. This increase was explained by a decrease in the mineral content of the solution leading to an aggregation of the proteins by electrostatic interactions [16]. Since EDBM acidification induced an important demineralization and reduction of the mineral content by more than 4 times in comparison with the control, there might have been the same kinds of aggregation in the WPI solution. Although the demineralization step that occurred with the EDBM acidification method seems to be the major factor in the selective precipitation of β-lg, it is in fact the presence of calcium that is known to promote the selective precipitation of one of the major whey proteins [16,21,39]. Indeed, the demineralization step leads to a general precipitation of proteins but also to a decrease in calcium concentration. Thereby, with a low conductivity, a low calcium content and a pH lower than 5, combined with a non-denaturing preheating treatment, β-lg tended to precipitate. This result was consistent with the results previously obtained by Bazinet et al. [21]. In this study, the use of EDBM acidification with a 10% WPI led to the production of an enriched fraction containing 97.3% of β-lg with a 53.4% recovery yield. This study was performed on a WPI obtained from thermal-treated milk named BiPro (Davisco Foods International Inc., MI). In our case, Prolacta is a WPI that comes from a non-denaturing process that allows proteins to stay in their native form with less than 4% denaturation. This major difference, coupled with the final pH value, explained why the β-lg purity was different between both studies. Proteins in BiPro may be more denatured from the beginning, and this denaturation may lead to a better aggregation and precipitation of the β-lg due to its unfolding. 

From another point of view, the promotion of β-lg precipitation may seem contradictory to observations performed by other studies. In fact, a decrease in calcium content generally promotes α-lac precipitation instead of β-lg [28,39,40]. The protein α-lac is influenced by calcium content since there is one mole of calcium by mole of α-lac in its center. The presence of a calcium ion in the protein promotes its stability in its globular tertiary structure. When α-lac loses its calcium ion, it results in the unfolding of the protein and its hydrophobic character. On the other hand, β-lg tends to be less influenced by calcium content according to those studies. For instance, Lucena et al. (2006) showed the influence of calcium concentration on a whey protein concentrate solution [39]. It appears that α-lac goes under its apo form when pH is close to its isoelectric point (pH 4.2–4.5), calcium concentration is low and temperature is between 40 and 60 °C. It also appears that β-lg is more denatured when a high calcium concentration is combined with extreme pH and temperature condition. Those observations are contradictory with those observed in our study, but it should be noted that one important factor differs between those studies, the total mineral content. Indeed, in studies demonstrating the precipitation of α-lac in low calcium concentration, the global mineral content was still high. In the study of Lucena et al. (2006), the mineral content was at least 29 times higher than the mineral content with EDBM acidification in our study, which means that only the calcium concentration changes [39]. In our study, the mineral content decreased with a decrease in calcium content, and that difference may explain why proteins did not react the same way. With a high global mineral content, the ionic strength stays high and keep proteins soluble by salting in phenomena. The majority of the studies on the influence of calcium on protein precipitation behavior were performed by adding CaCl_2_, for instance, in whey protein solution [28,39,40]. Salting in and salting out phenomena did not occur under the same condition when total mineral content was decreased. 

Protein concentration also plays an important role in the recovery yield when the impact of mineral concentration is considered [16,21]. The concentration of protein in the solution used in our study was higher than the one used in Amundson et al.’s (1982) [16] study, and that can lead to an increase of the aggregation, as shown by Bazinet et al. (2004) [21]. This last study showed that the maximal recovery yield was obtained for a protein solution containing 10% whey proteins in comparison with 5% or 20%. In their study, they also compared their process with a chemical acidification that consisted of an addition of 1.0 N HCl. With EDMB, they succeeded in recovering 54% of the total protein, while only 14.4% of the total protein was precipitated with chemical acidification. Those results were consistent with the results obtained in our study. The demineralization step occurring with the EDBM acidification leads to a higher total protein recovery yield, but this factor combined with the selective precipitation of β-lg previously presented leads to a higher β-lg recovery yield. Indeed, when a low mineral content is combined with a low protein content, no effect is observed. However, when a low mineral content is combined with a high protein content, like in our case with a 10% protein content, the impact of the mineral content is important. Decreasing the mineral content means less mineral around the protein to create the hydration layer and leads to the exposure of protein charged zones. Their aggregation and precipitation may occur. Indeed, at a pH between 6.6 and 5.1, α-lac and β-lg are negatively charged, but when the pH is close to their isoelectric point, the protein charge is neutral and there is no more electrostatic repulsion between proteins, leading consequently to their aggregation. The lack of minerals will then promote precipitation of the aggregates [3]. 

##### Effect of Temperature

The choice of adding a preheating treatment to the process initially came from the different denaturation rate between the WPI used in our study from the one used in Bazinet et al. (2004) [21] that allowed high β-lg recovery yield and purity as previously discussed. This difference in initial denatured protein contents can lead to different precipitation comportment since denaturation of whey protein leads to the formation of a complex through thiol–disulfur interchange reactions [21,28]. By applying temperature close to the denaturation temperature of α-lac and β-lg, it was expected to obtain similar yields to the one of Bazinet et al. (2004) [21]. As presented with the protein purity, recovery yield and β/α ratio, it was shown that the preheating treatments tested influenced the composition and the weight of the precipitated fractions. Literature mostly presents the impact of a heating treatment on an acidified solution. However, proteins can lead to different comportment since they can return or not to their initial state according to the thermal treatment used. Thermal denaturation of proteins occurs in two steps. The first step consists of a noncovalent alteration of protein, while the second step is the irreversible aggregation that may lead to precipitation. For this second step, if the thermal energy is too high, covalent bonds can be broken and then result in a thermal degradation [28]. Temperatures used in the present study were lower than the denaturation temperature of both proteins (65 °C for α-lac and 75 °C for β-lg [7]), so it is possible that only the first step of denaturation occurred. In fact, using nonpermanent denaturing temperature under 65 °C allows keeping α-lac under its metal form (with calcium ions), which means that α-lac is not denatured under these conditions. Indeed, heating WPI solution at its physiological condition with temperature up to 60 °C did not allow α-lac to lose its calcium ion and thereby to be denatured, as demonstrated by Croguennec et al. (2008) [7], as it would have been if the heating treatment was carried out on the acidified solution. On the other hand, β-lg has a higher denaturation temperature but is less stable when subjected to heating treatment [3]. 

The comparison of chemical acidification and the control showed that preheating treatment at 60 °C promotes the precipitation of α-lac. This result was consistent with the results previously obtained by Pearce et al. (1983) [10] that reported the precipitation of α-lac from different whey protein solutions under heat treatment from 55 to 70 °C when solutions were acidified with HCl. The conditions produced by a chemical acidification without demineralization and combined with a gentle heat treatment, around its denaturation temperature of 65 °C, promote α-lac precipitation at the expense of β-lg. However, since in our study the proteins were not from a model solution, only trends can be observed during chemical acidification with a slight increase in α-lac purity in the precipitate when a preheating treatment was carried out. It was also expected to obtain no selective precipitation of α-lac or β-lg with chemical acidification without preheating treatment (20 °C) since none of the proteins were in conditions promoting their precipitation. However, it was expected to obtain higher β-lg purity rates in the EDBM-acidified samples than in chemically acidified samples. The use of preheating temperatures of 50, 55 and 60 °C increased the β-lg purity for the EDBM acidification, due to the mechanism of β-lg thermal denaturation. Naturally, at 20 °C the β-lg stays in its native dimer form, but when heated between 40 and 55 °C, β-lg dimer turns into two monomers. When heated at 60 °C, the monomers tend to turn into the R-state, which is obtained by intramolecular transition that affects the α-helix and masks the free thiol group that is normally hidden in the core of the protein. This makes the reactive cysteine more accessible and can result in polymerization of the β-lg [3,41]. This phenomenon, combined with a low conductivity, can lead to the precipitation of β-lg aggregates promoted by the lack of salt. 

The combination of pH, mineral content and temperature appears to play an important role in the selective separation of β-lg and α-lac from WPI solution. In fact, the condition of EDBM acidification method combined with a preheating temperature of 60 °C allowed the highest recovery yield with the highest β-lg purity. However, as presented, a pH of 4.65 instead of 4.8 could have allowed higher yield. The maximal temperature of 60 °C for the preheating treatment also seems to be a major limitation for this process since it did not allow denaturation of the whey protein. The use of higher temperatures could also have allowed different results. 

To confirm the hypothesis, some tests were carried out with the same EDBM system in the same conditions but with a heating treatment at 60 and 80 °C for 20 min after acidification. For those tests, precipitate weight, total protein content and protein profile determination with SDS-PAGE electrophoresis gel were carried out. The first observation was that heating after acidification promotes precipitation of total protein, since it allows recovering 56.2 ± 2.2% and 85.1 ± 2.0% of total proteins for the 60 and 80 °C heating treatments, respectively. However, β-lg purity in the precipitate fraction was lower than the one obtained by heating treatment before acidification. The fraction tested at 60 °C has a β-lg purity of 64.6 ± 2.1%, and the one at 80 °C has a β-lg purity of 67.4 ± 0.3%. This means that, in those conditions, there was the promotion of α-lac precipitation that corresponded to the literature. With the use of EDBM acidification, higher purities were obtained when heating treatment was performed before acidification, but recovery yields were higher when performed after acidification.

## 4. Materials and Methods 

### 4.1. Material 

Whey protein isolate (WPI) powder (Prolacta 95; batch 672163 04:16:25 008788) was kindly provided by Lactalis (Retiers, France). According to the manufacturer, the total protein, fat, lactose and mineral contents were 95%, 0.4%, 3.0% and 3.0%, respectively. 

### 4.2. EDBM Cell Configuration

**Figure 4:**
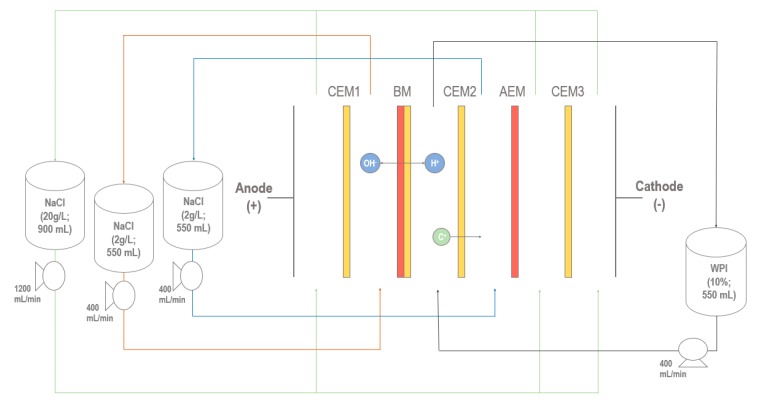
EDBM cell configuration for the electrochemical acidification of whey protein solution.

An electroacidification cell (MP type, 100 cm^2^ effective surface area) manufactured by ElectroCell Systems AB Co. (Täby, Sweden) was used with three cationic membranes (CEM, Astom, Tokyo, Japan), one anionic membrane (AEM, Astom, Tokyo, Japan) and one bipolar membrane (BM, Astom, Tokyo, Japan) (Figure 4). This configuration was formed by four closed loops, where whey protein solution, recovery solutions (NaCl 2 g/L) and electrode rinsing solution (NaCl 20 g/L) were circulated with centrifugal pumps (CL3503, Baldor Electric Company, Arkansas, USA) and their flow rates controlled at 400 mL/min by flowmeters (F-550, Blue-White Industries Ltd., CA, USA). The ion-exchange membrane allows the migration of anions or cations, while the bipolar membrane allows the production of H^+^ and OH^–^ into the whey protein solution and the NaCl 2 g/L recovery compartment, respectively. The volume of the WPI solution (550 mL) was the same as both compartments of NaCl 2 g/L during all the acidification. This configuration allowed the simultaneous acidification and demineralization of the WPI solution and was chosen to minimize minerals and protein fouling of the membranes, based on recent work of Mikhaylin et al. [19].

### 4.3. Experimental Protocol

The WPI powder was rehydrated overnight at 4 °C in MilliQ water to produce a 10% protein solution. This solution was then heated at 20, 50, 55 or 60 °C for 20 min under agitation and then centrifuged at 10,000× *g* for 20 min at 20 °C to remove insoluble particles. The choice of temperature was based on preliminary tests (results not shown) performed on EDBM system showing that temperatures above 65 °C produce an instantaneous membrane fouling in the whey solution compartment that limits the process efficiency. 

After centrifugation, 550 mL of the supernatant was acidified either by chemical acidification or by electrochemical acidification using EDBM to a pH of 4.8 which has also been determined by preliminary tests (results not shown) showing that, in the conditions used for electrochemical acidification in this study and without salt addition, it was not possible to decrease the pH under 4.8, as explained by Bazinet et al. (2004) [22]. For chemical acidification, 2.0 N HCl was added directly to the protein solution under a constant agitation. Conductivity and pH of the WPI solution were recorded throughout the acidification step for both methods. For electrochemical acidification, the duration, the cell resistance and intensity were also recorded every 5 min. The voltage during EDBM was maintained constant at 20 V. New membranes were used for each run. 

**Figure 5:**
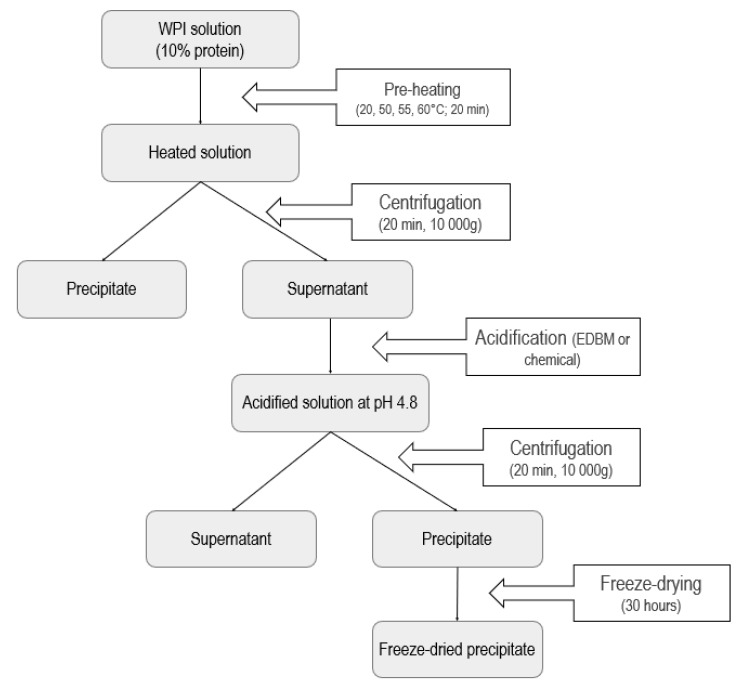
Main stages of the experimental protocol.

At the end of each acidification, 450 mL of the acidified WPI solution was centrifuged for 20 min at 10,000× *g*, at 20 °C. The precipitate was recovered and freeze-dried (Model Freezone 4.5, Labconco, Kansas City, MI, USA) (see Figure 5). Isolate powders were weighed, ground and stored at 4 °C before the determination of proximate composition as well as protein profiles. All analyses were performed on the final freeze-dried precipitate of each condition in comparison with the unheated and unacidified WPI (control). Every combination of acidification and temperature was performed in triplicate. 

### 4.4. Electrochemical Acidification Parameters

#### 4.4.1. System Resistance

The EDBM system resistance (*R*, in Ω) was calculated, using Ohm’s Law, from the voltage (*U*, in V) and the current intensity (*I*, in A) read directly from the indicators on the power supply provided by B&K Precision (Model 9110, Yorba Linda, CA, USA).

(1) $$R=\frac{U}{I}$$

#### 4.4.2. Membrane Electrical Conductivity 

Each membrane used for EDBM acidification was characterized before and after each experiment. To do so, the membranes were soaked for 30 min in a 0.5 M NaCl solution before measurement of their thickness and conductance (*G*) allowing the calculation of the electrical conductivity. A 10-mm-diameter flat contact point electronic digital micrometer, from Marathon Watch Company Ltd. (Richmond Hill, ON, Canada) was used to measure the membrane thickness. Six measurements at different locations on the effective surface of the membrane were taken to determine the average thickness (in cm) [42].

Conductance was obtained by a YSI conductivity instrument Model 3100 (Yellow Springs, OH, USA) equipped with a specially designed cell as described by Cifuentes-Araya [42]. For this analysis, six measurements were taken on different locations on the effective surface of the membrane in the reference solution (*G_m + S_*), and six others were taken with only the reference solution (*G_s_*), 0.5 M NaCl solution. Conductance of the reference solution combined with the conductance of the membrane in the reference solution allow determination of the electrical resistance of the membrane (*R_m_*) according to Equation (2).

(2) $$R_{m}=\frac{1}{G_{m}}=\frac{1}{G_{m+s}}-\frac{1}{G_{s}}=R_{m+s}-R_{s}$$

Once the thickness (*L*) and the electrical resistance of the membrane (in Ω) were determined, the electrical conductivity (*k*) was calculated according to Equation (3). *A* is the electrode area (1 cm^2^).

(3) $$k=\frac{L}{R_{m}A}$$

### 4.5. Whey Protein Solution Parameters

#### 4.5.1. Conductivity

Conductivity of protein solutions was measured during both acidification methods. To do so, a YSI conductivity instrument Model 3100 (Yellow Springs, OH, United States) equipped with an automatic temperature compensation (ATC) immersion probe (Model 3252, cell constant K = 1/cm) was used. For electrochemical acidification, the demineralization rate (DR, in %) of the whey protein solution was determined according to Equation (4).

(4) $$DR=\left(1-\frac{k_{t}}{k_{0}}\right)\times 100$$

*k_t_* is the solution conductivity at time *t* and *k*_0_ is the solution conductivity at time = 0 [43].

#### 4.5.2. pH Evolution during Acidification

The pH of WPI solutions during chemical and electrochemical acidification was measured by a pH meter equipped with a special electrode for complex solutions from ThermoFisher Scientific Orion (Model AquaPro pH Combination Electrodes).

### 4.6. Precipitate Analyses 

#### 4.6.1. Proximate Composition 

All the analyses of proximate composition were performed on the freeze-dried precipitates and on the control, which is the unheated and unacidified WPI. Fat content was determined according to the AOAC Official Methods of Analysis Nos. 974.09 and 989.05 [44]. Lactose concentration was determined through high-performance liquid chromatography (HPLC) as described by [45]. Samples were prepared following the ISO 22662:2007 and IDF 198:2007 (ISO/IDF, 2007) method [46]. Moisture and ash content were determined according to the methods 927.05 and 930.30, respectively [47]. Calcium, potassium, magnesium, sodium and phosphorous concentrations were obtained by ICP-OES (Model Agilent 5110 SVDV, Agilent Technologies, Mulgrave, VIC, Australia) using the following wavelengths (nm): 393.366; 396.847; 422.673 (Ca), 766.491 (K), 279.553; 280.270; 285.213 (Mg), 588.995; 589.592 (Na), 177.434; 178.222; 213.618; 214.914 (P). Total nitrogen was determined by the Dumas combustion method using a Rapid Micro N Cube (Elementar, Francfort-sur-le-Main, Germany) and was converted into a protein concentration by applying a 6.38 conversion factor [16]. 

#### 4.6.2. Protein Yield

##### Protein Profiles

Protein profiles of precipitates and control were determined by mono-dimensional SDS-PAGE. Firstly, solutions of 0.7 mg of protein/mL of each sample were prepared. A volume of 25 µL of these solutions was mixed with 25 µL of the sample buffer (5% 2-mercaptoethanol, 95% Laemmli buffer). Samples were then boiled for 5 min before being cooled in an ice bath. Ten microliters of each solution (precipitates, control, as well as α-lactalbumin and β-lactoglobulin standard) and 5 µL of molecular weight control (Precision Plus Protein All Blue Prestained Protein Standards, Bio-Rad) were loaded into Mini-Protean TGX Stain-Free gels from Bio-Rad containing 12% polyacrylamide (Bio-Rad, Hercules, CA, USA). Reducing electrophoresis was stopped when samples were at 1 cm from the bottom of the gel. Migration occurred in a running buffer (10% Tris-glycine SDS Buffer, 90% deionized water). After migration, gels were stained with Coomassie Brilliant Blue solution (1% Coomassie Brilliant Blue, 10% ethanol, 10% acetic acid and 79% deionized water) for 60 min and then destained with four 30-min treatments in a fading solution (10% methanol, 10% acetic acid and 80% deionized water). Pictures of the gel were obtained by using Gel Doc XR from Bio-Rad. Quantification of the α-lac and β-lg bands obtained on the gel was performed with Image Lab 6.0.1 software by Bio-Rad. This quantification allowed determination of their purity in the precipitate fraction.

##### Protein Recovery Yield

From the purity rate (P_x_) of the precipitate obtained combined with the total protein content (T), it was possible to determine the recovery yield (R_x_) of α-lac and β-lg using Equations (5) and (6).

(5) $$R_{\alpha -\mathrm{lac}}=\frac{P_{\alpha -\mathrm{lac}}\left(X_{p}\times \;T\right)}{P_{c\;\alpha -\mathrm{lac}}\times {\;X}_{\mathrm{tp}}}$$

or

(6) $$R_{\beta -\mathrm{lg}}=\frac{P_{\beta -\mathrm{lg}}\left(X_{p}\times \;T\right)}{P_{c\;\beta -\mathrm{lg}}\times {\;X}_{\mathrm{tp}}}$$

where X_p_ is the quantity of freeze-dried precipitate (g) obtained, P_c x_ is the purity rates of the control and X_tp_ is the 45 g of protein that can be recovered knowing that the final precipitate comes from a 450 mL sample with a protein content of 10%.

### 4.7. Statistical Analyses

All samples were prepared and analyzed in triplicate. Data obtained were reported as mean value ± standard deviation. One-way and two-way ANOVA tests were performed to determine significant differences and interactions between the two main factors, namely the preheating temperature and the acidification method. Data that presented a failed normality test were transformed using 1x or lnx. Statistical differences between the conditions were analyzed by Tukey test with a *p*-value of <0.05. The statistical analyses of membranes and WPI solution conductivity before and after EDBM treatment were performed using *t*-tests. SigmaPlot software (version 12, Systat Software, San Jose, CA, USA) was used for all statistical analyses.

## 5. Conclusions

These results show that the use of a preheating temperature of 60 °C applied on a 10% undenatured whey protein solution combined with acidification and demineralization with EDBM provides a β-lg-enriched fraction with a purity of 81.75 ± 0.33% and a recovery yield of 35.78 ± 4.38%. Chemical acidification did not allow to obtain such an enriched fraction either in the precipitate or in the supernatant. The use of a 60 °C preheating temperature also allows an increase in the recovery yield of the process by allowing a greater precipitation of the proteins. Meanwhile the ash, lactose, lipid and total protein contents were the same regardless of the temperature used. Samples produced in all these conditions had the same amount of proteins, but the protein profile (purity) and the precipitate weight (recovery yield) differed depending on the conditions used. Meanwhile, membrane conductivity, duration, solution conductivity and pH were not affected by the preheating temperature during the EDBM treatment. This means that preheating temperature up to 60 °C allows better separation without causing fouling problems.

Further works will be aimed at increasing purity and recovery yields of both fractions. To do so, salt will be added to the 10% whey protein solution in a way to continue the acidification process with the EDBM system to a pH of 4.65 as demonstrated by Bazinet et al. [22]. Studies on separation of whey proteins have already demonstrated the interest of using a pH value of 4.65 to specifically precipitate β-lg from complex solution when combined with a demineralization step [16,21]. The determination of an optimal denaturation rate of the protein will allow determining ideal WPI to obtain selective separation of the main whey proteins. With the aim of separating β-lg and α-lac with high purity and recovery yield, it is recommended to continue studies with heating treatment before acidification or to change the WPI used, using WPI that is not composed of native protein for the WPI solution. In fact, if the WPI undergoes heating stages during its production, this separation process can lead to higher purity and recovery yields without having to add a preheating step. Once the selective separation is obtained, the α-lac fraction may be used in infant formula, and the β-lg fraction may be used as functional ingredients. Acidification and demineralization obtained by EDBM lead to a decrease in protein solubility that can have an impact on functional properties of proteins [16,21], since the pH of the final fraction was close to their isoelectric point; this solubility can be recovered by adjusting the pH and ionic strength of the final precipitate [48,49]. As demonstrated previously by Masson et al. [45] on caseins, the adjustment of the EDBM-precipitated fraction (acid casein) to its initial pH allows a full recovery of its solubility (caseinate). However, evaluation of the fraction functionalities and activities after EDBM acidification and demineralization will be necessary.

## Figures and Tables

**Table 1:** Membrane conductivity before and after electrodialysis with bipolar membrane (EDBM) treatment regardless of the temperature used (placed in the same order as stacked in the system configuration).

Membrane	Conductivity (mS/cm)		Variation (%)
	Before	After	
**CEM 1**	8.6 ± 0.3 ^a*^	8.1 ± 0.5 ^a^	−5.9 ± 6.0 ^B^
**BM**	5.1 ± 0.1 ^b^	4.8 ± 0.1 ^a^	−5.3 ± 3.1 ^B^
**CEM 2**	8.5 ± 0.3 ^b^	5.6 ± 0.1 ^a^	−34.5 ± 2.6 ^C^
**AEM**	5.0 ± 0.1 ^a^	5.2 ± 0.1 ^b^	4.2 ± 2.4 ^A^
**CEM 3**	8.6 ± 0.2 ^b^	7.9 ± 0.4 ^a^	−8.6 ± 5.6 ^B^

* Data with different letters (a, b or A, B) are significantly different; lowercase letters indicate differences between conductivities, before and after EDBM treatment, for the same membrane; uppercase letters indicate differences between membranes.

**Table 2:** Composition of final precipitates on dry basis in comparison with the control (g/100 g dry powder).

	EDBM				Chemical Acidification				Control **
	20 °C	50 °C	55 °C	60 °C	20 °C	50 °C	55 °C	60 °C	
**Proteins**	97.56 ± 1.00 ^ab, A^ *	96.89 ± 0.52 ^ab, A^	97.19 ± 0.36 ^ab, A^	97.59 ± 1.32 ^ab, A^	98.04 ± 0.78 ^ab, A^	98.26 ± 0.64 ^ab, A^	97.85 ± 0.21 ^ab, A^	98.69± 0.65 ^b, A^	96.31 ± 0.11 ^a^
**Ashes**	0.45 ± 0.02 ^a, B^	0.43 ± 0.02 ^a, B^	0.43 ± 0.01 ^a, B^	0.37 ± 0.03 ^a, A^	1.03 ± 0.04 ^b, B^	1.02 ± 0.02 ^b, B^	0.99 ± 0.04 ^b, AB^	0.94 ± 0.01 ^b, A^	1.83 ± 0.06 ^c^
**Ca**	0.041 ± 0.010 ^a, A^	0.056 ± 0.013 ^a, A^	0.058 ± 0.009 ^a, A^	0.039 ± 0.003 ^a, A^	0.258 ± 0.020 ^b, A^	0.267 ± 0.005 ^bc, A^	0.270 ± 0.006 ^bc, A^	0.304 ± 0.005 ^c, B^	0.660 ± 0.034 ^d^
**K**	0.018 ± 0.013 ^a, A^	0.019 ± 0.011 ^a, A^	0.020 ± 0.012 ^a, A^	0.011 ± 0.010 ^a, A^	0.107 ± 0.005 ^b, A^	0.127 ± 0.013 ^bc, AB^	0.141 ± 0.007 ^bc, B^	0.150 ± 0.014 ^c, B^	0.276 ± 0.023 ^d^
**Mg**	0.009 ± 0.010 ^a, A^	0.005 ± 0.002 ^a, A^	0.012 ± 0.006 ^a, A^	0.002 ± 0.000 ^a, A^	0.031 ± 0.008 ^b, A^	0.038 ± 0.001 ^b, AB^	0.039 ± 0.001 ^b, AB^	0.043 ± 0.001 ^b, B^	0.098 ± 0.004 ^c^
**Na**	0.108 ± 0.121 ^ab, A^	0.035 ± 0.012 ^a, A^	0.164 ± 0.063 ^ab. A^	0.021 ± 0.014 ^a, A^	0.124 ± 0.066 ^ab, A^	0.095 ± 0.011 ^a, A^	0.105 ± 0.032 ^ab, A^	0.119 ± 0.009 ^ab, A^	0.259 ± 0.043 ^b^
**P**	0.152 ± 0.085 ^abc, A^	0.129 ± 0.043 ^ab, A^	0.211 ± 0.023 ^bc, A^	0.066 ± 0.014 ^a, A^	0.460 ± 0.020 ^f, C^	0.453 ± 0.009 ^f, C^	0.421 ± 0.002 ^ef, B^	0.333 ± 0.011 ^de, A^	0.245 ± 0.009 ^cd^
**Lipids**	0.19 ± 0.01 ^a, A^	0.16 ± 0.03 ^a, A^	0.19 ± 0.02 ^a, A^	0.18 ± 0.01 ^a, A^	0.16 ± 0.02 ^a, A^	0.18 ± 0.02 ^a, A^	0.18 ± 0.02 ^a, A^	0.14 ± 0.01 ^a, A^	0.16 ± 0.01 ^a^
**Lactose**	0.16 ± 0.01 ^a, A^	0.20 ± 0.02 ^ab, B^	0.20 ± 0.01 ^ab, B^	0.20 ± 0.01 ^ab, B^	0.21 ± 0.01 ^b, A^	0.24 ± 0.02 ^b, A^	0.24 ± 0.02 ^b, A^	0.30 ± 0.01 ^c, B^	0.77 ± 0.01 ^d^
**Total**	98.35 ± 1.04 ^ab^	97.68 ± 0.59 ^a^	98.01 ± 0.41 ^ab^	98.34 ± 1.37 ^ab^	99.44 ± 0.84 ^ab^	99.71 ± 0.70 ^ab^	99.26 ± 0.29 ^ab^	100.07 ± 0.69 ^b^	99.07 ± 0.18 ^ab^

* Data in the same line with different letters (a, b or A, B) are significantly different; lowercase letters indicate differences between all the data on the line; uppercase letters indicate differences between temperature within EDBM or CA for the same line; ** unheated and unacidified WPI.

**Table 3:** Protein purity, weight of protein and recovery yield in the precipitate fractions recovered after acidification at pH 4.8.

		EDBM				Chemical Acidification				Control
		20 °C	50 °C	55 °C	60 °C	20 °C	50 °C	55 °C	60 °C	
**Purity (%)**	β-lg	78.32 ± 0.68 ^c, A^*	82.25 ± 1.60 ^d, B^	82.28 ± 0.26 ^d, B^	81.75 ± 0.33 ^d, B^	74.12 ± 0.80 ^a, BC^	74.13 ± 0.63 ^a, C^	72.29 ± 0.79 ^ab, AB^	70.94 ± 0.55 ^b, A^	74.23 ± 0.24 ^a^
	α-lac	21.68 ± 0.68 ^b, B^	17.75 ± 1.60 ^a, A^	17.72 ± 0.26 ^a, A^	18.43 ± 0.33 ^a, A^	25.88 ± 0.80 ^c, AB^	25.87 ± 0.63 ^c, A^	27.71 ± 0.79 ^cd, BC^	29.06 ± 0.55 ^d, C^	25.77 ± 0.24 ^c^
**Weight of protein (g)**	β-lg	3.95 ± 0.21 ^bc, A^	5.52 ± 0.42 ^cd, AB^	6.38 ± 0.56 ^d, B^	11.95 ± 1.46 ^e, C^	1.48 ± 0.05 ^a, A^	1.90 ± 0.01 ^a, AB^	2.24 ± 0.01 ^a, B^	3.87 ± 0.49 ^b, C^	33.40 ± 0.11 ^f^
	α-lac	1.09 ± 0.02 ^bcd, A^	1.20 ± 0.21 ^cde, A^	1.37 ± 0.13 ^de, A^	2.70 ± 0.32 ^f, B^	0.52 ± 0.02 ^a, A^	0.66 ± 0.03 ^ab, AB^	0.85 ±0.03 ^abc, B^	1.59 ± 0.24 ^e, C^	11.59 ± 0.11 ^g^
	Ratio β/α	3.62 ± 0.14 ^b, A^	4.66 ± 0.49 ^c, B^	4.64 ± 0.08 ^c, B^	4.43 ± 0.10 ^c, B^	2.87 ± 0.12 ^a, A^	2.87 ± 0.10 ^a, A^	2.62 ± 0.10 ^a, B^	2.44 ± 0.06 ^a, B^	2.88 ± 0.04 ^a^
	Total protein	5.05 ± 0.23 ^bc, A^	6.72 ± 0.61 ^cd, AB^	7.75 ± 0.68 ^d, B^	14.65 ± 1.78 ^e, C^	1.99 ± 0.05 ^a, A^	2.57 ± 0.04 ^a, AB^	3.10 ±0.04 ^ab, B^	5.47 ± 0.73 ^c, C^	45.00 ± 0.00 ^f^
**Recovery yield (%)**	β-lg	11.83 ± 0.64^c, A^	16.53 ± 1.24 ^cd, AB^	19.09 ± 1.67 ^d, B^	35.78 ± 4.39 ^e, C^	4.42 ± 0.14 ^a, A^	5.70 ± 0.03 ^a, AB^	6.70 ± 0.04 ^ab, B^	11.60 ± 1.46 ^bc, C^	-
	α-lac	9.42 ± 0.19 ^bcd, A^	10.33 ± 1.80 ^cde, A^	11.85 ± 1.12 ^de, A^	23.27 ± 2.76 ^f, B^	4.44 ± 0.15 ^a, A^	5.73 ± 0.22 ^ab, AB^	7.40 ± 0.30 ^abc, B^	13.72 ± 2.08 ^e, C^	-
	Total protein	11.21 ± 0.52 ^bc, A^	14.93 ±1.35 ^cd, AB^	17.22 ± 1.52 ^d, B^	32.56 ± 3.96 ^e, C^	4.42 ± 0.12 ^a, A^	5.71 ± 0.08 ^a, AB^	6.88 ± 0.09 ^ab, B^	12.15 ± 1.62 ^c, C^	-

* Data in the same line with different letters (a, b or A, B) are significantly different; lowercase letters indicate differences between all the data of β-lg, α-lac, ratio or total protein for the same line; uppercase letters indicate differences between temperature within EDBM or CA for the same line.
